# Molecular Mechanism and Health Role of Functional Ingredients in Blueberry for Chronic Disease in Human Beings

**DOI:** 10.3390/ijms19092785

**Published:** 2018-09-16

**Authors:** Luyao Ma, Zhenghai Sun, Yawen Zeng, Mingcan Luo, Jiazhen Yang

**Affiliations:** 1 Economics and Management College, Southwest Forestry University, Kunming 650224, China; ma403294928@163.com; 2 Key Laboratory for Forest Resources Conservation and Utilisation in the Southwest Mountains of China, Southwest Forestry University, Ministry of Education, Kunming 650224, China; lmc1961@sohu.com; 3 Biotechnology and Germplasm Resources Institute, Yunnan Academy of Agricultural Sciences, Kunming 650205, China; yangjiazhen415@163.com

**Keywords:** blueberry, functional ingredients, chronic disease, functional food

## Abstract

Functional ingredients in blueberry have the best health benefits. To obtain a better understanding of the health role of blueberry in chronic disease, we conducted systematic preventive strategies for functional ingredients in blueberry, based on comprehensive databases, especially PubMed, ISI Web of Science, and CNKI for the period 2008–2018. Blueberry is rich in flavonoids (mainly anthocyanidins), polyphenols (procyanidin), phenolic acids, pyruvic acid, chlorogenic acid, and others, which have anticancer, anti-obesity, prevent degenerative diseases, anti-inflammation, protective properties for vision and liver, prevent heart diseases, antidiabetes, improve brain function, protective lung properties, strong bones, enhance immunity, prevent cardiovascular diseases, and improve cognitive decline. The anthocyanins and polyphenols in blueberry are major functional ingredients for preventive chronic disease. These results support findings that blueberry may be one of the best functional fruits, and further reveals the mechanisms of anthocyanins and polyphenols in the health role of blueberry for chronic disease. This paper may be used as scientific evidence for developing functional foods, nutraceuticals, and novel drugs of blueberry for preventive chronic diseases.

## 1. Introduction

Berries are the best dietary sources of health benefits, these benefits are associated with their specific chemical and biological properties, such as Rosaceae Rubus (raspberry and blackberry), Fragaria (strawberry), and Ericaceae Vaccinium (blueberry and cranberry), major bioactive compounds contain anthocyanins, phenolic acids, ascorbic acid, flavonols, and tannins [1]. Berry anthocyanidins (cyanidin, malvidin, peonidin, petunidin, and delphinidin) have various anticancer effects [2] however, cyanidin and peonidin have similar anticancer effects to that of black rice [3]. Berry meals should be used as an important source of phenolics for human health, especially the decreasing order of the total phenolics, scavenging, and chelating capacity for blackberry > black raspberry > blueberry [4]. Berries (strawberry, raspberry, blackberry, and blueberry) have high total phenolic compounds, but significant differences in antioxidant capacity, i.e., blackberries and strawberries had higher anthocyanin content, meanwhile, the availability of total phenolic compounds was higher than anthocyanins (33–73%) [5]. The concentrations of bioflavonoids in commercial berry juices (blackcurrant blueberry, red raspberry, and cherry) (mg/100 g of juice) are as follows. Luteolin-7-*O*-glucoside (5.6–10.2) > rutin (0.4–6.5) > quercetin (0.21–5.12) > kaempferol (0.05–1.2) > kaempferol-3-*O*-glucoside (0.02–0.12), however blackcurrant > blueberry > red raspberry > cherry for total flavonoids; the antioxidant activity of juice extracts is expressed as IC50 values from 8.56 to 14.05 mg/kg [6]. The total anthocyanin and polyphenol content in blueberry juice was higher, which also has higher antioxidant activity, but cranberry juice had a greater capacity than blueberry juice as an α-glucosidase inhibitor [7].

Blueberry is one of the most nutritious foods and cultivated worldwide. The global total output of blueberry was 629,720 tons in 2016; major distributors of the blueberry in the world are USA, Chile, Canada, Spain, China, Morocco, and 30 other countries. The production of natural food pigments continues to grow worldwide with an annual growth rate of 6.22% from 2015 to 2019, especially five pigments (anthocyanins, carotenoids, betalains, and chlorophylls) which have been used to color foods [8]. Blueberry with abundant anthocyanins can improve vision, is anticancer, antidiabetes, anti-obesity, is preventive against neurodegeneration and macular degeneration as well as osteoporosis, can reduce hyperlipidemia and hypertension, as well as heart disease through its apoptosis, antioxidant, anti-inflammation, and anti-angiogenesis effects [9]. Blueberries with 15,886 expressed sequence tag-SSR (EST-SSR) loci were identified and 7705 simple sequence repeats (SSRs) of adequate flanking sequence were found [10]. Distinct anticarcinogenic effects associated with the different phytochemical signatures of blueberry (downregulating CYP1A1 expression), and black raspberry (downregulated ERα expression) [11]. Blueberry and mulberry juice can have anti-obesity properties based on being able to reduce insulin resistance and lipid accumulation and lower serum cholesterol and leptin secretin [12]. Blueberry juice and bifidobacteria improve nonalcoholic fatty liver disease by activating SIRTI-mediating signaling pathway [13].

## 2. Functional Ingredients in Blueberry

The bioactive components in blueberry include anthocyanins, polyphenols, and antioxidant properties. Pectin from blueberry powder included cyanidin-3-glucoside (C_21_H_21_O_11_Cl) and anthocyanidins, however anthocyanidins including cyanidin (color change with pH, red < 3, violet 7–8, blue > 11), pelargonidin (C_15_H_11_O_5_^+^, orange), malvidin (blue), petunidin (dark-red, purple), and delphinidin (blue, blue-red), which may represent two major mechanisms of anthocyanin stacking and ionic interaction for pectin and anthocyanin binding [14]. Blueberries are extraordinarily rich in polyphenols, including 3-glucoside/arabinoside/galactoside-based polymers of delphinidins (C_15_H_11_O_7_^+^), petunidins [C_16_H_13_O_7_^+^(Cl^−^)], peonidins (C_16_H_13_O_6_^+^), malvidins (C_17_H_15_O_7_), and cyanidins (C_15_H_11_O_6_^+^) [15]. The antioxidant properties of blueberry juices, total phenolic content (0.85–2.81 mg/g), quinic (C_7_H_12_O_6_; 0.203–3.614 mg/g) and chlorogenic acids (C_16_H_18_O_9_; 0.02–0.347 mg/g), and rutin (C_27_H_30_O_16_; 0.00–26.88 μg/g) have lager variation, however ABTS(+) scavenging capacity (6.38–20.9 μmol/g), ferric reducing antioxidant power (3.07–17.8 μmol/g), and oxygen radical absorbance capacity (4.21–45.68 μmol/g) [16] have less variation. Oral anthocyanins that go through the blood–brain barrier can improve visual function by increasing rhodopsin regeneration [9]. The detection limits (μg/L) of succinic (C_4_H_6_O_4_, 2.5), citric (C_6_H_8_O_7_, 2.0), salicylic (C_7_H_6_O_3_, 3.4), malic (C_4_H_6_O_5_, 1.5), benzoic (C_6_H_5_COOH, 2.2), sorbic (C_6_H_8_O_2_, 0.8), ascorbic (C_6_H_8_O_6_, 1.5), and tartaric acid (C_4_H_6_O_6_, 4.5) in blueberry juices have been developed [17]. Colonic fermentation of wild blueberry polyphenols are degraded to syringic (C_9_H_10_O_5_), cinnamic (C_9_H_8_O_2_), caffeic (C_9_H_8_O_4_), and protocatechuic acids (C_7_H_6_O_4_) [18]. Blueberry (*Vaccinium uliginosum* L.) from the Chinese Changbai Mountains contained five main components (delphinidin 40.70%, cyanidin 3.40%, petunidin 17.70%, peonidin 2.90%, and malvidin 35.50%), which could be a promising functional food to suppress colorectal cancers [19]. A Brazilian blueberry had very high content of total phenolics (16.22–34.57 g/kg), total anthocyanins (1.4–3.18 g/kg), and carotenoids [20]. Malvidin derivatives of blueberry had lower antioxidant capacity than that of blackberry; blackberry had the highest total antioxidant capacity which is associated with cyanidin-3-*O*-glucoside, which accounted for 94% of blackberry anthocyanins [21]. The juice obtained from Pulsed Electric Field pretreated blueberry had a significantly higher antioxidant activity (+31%), total phenolic (+43%), and anthocyanin content (+60%) [22].

## 3. Preventive Chronic Disease of Blueberry

### 3.1. Anticancer and Functional Ingredients of Blueberry

Blueberry is a good source of anthocyanins and phenolic acids (see Table 1), which showed, not only higher tumor stayer and lower proliferative, antiapoptotic, and angiogenic transcript levels [15], but also prevented carcinogenesis and reduced the risks of cancer recurrence [23] as well as having potential as a radiosensitizer for treating cervical cancer [24]. Blueberry anthocyanins and their production with pyruvic acid (C_3_H_4_O_3_) can slow the progress of cancer by inhibiting the proliferation of cancer cells [25]. Blueberry anthocyanin ranged from 1.02 to 1.95 g/kg of malvidin-3-glucoside for fresh weight, which has antiproliferative and apoptotic properties in cancer cells and are used as chemopreventive metastasis [26], however its anticancer ability using HepG-2 cells is associated with the concentration of anthocyanins [27]. Mitochondria benefits from the protective effects of blueberry anthocyanin extract against acrylamide toxicity [28]. Pterostilbene in blueberries can control worsened/obdurate myeloma therapy and major therapeutic myeloma of bortezomib chemotherapy [29]. Lowbush blueberry proanthocyanidins can enhance apoptosis induction in human colorectal cancer cell lines, which represents important nutritional chemoprevention of colorectal cancer [30]. The dietary food for blueberry husks with probiotics can delay colonic cancer [31]. Blueberry juice has similar anti-premutagenic activity to vitamin C, and also acts a methylation suppressor for methylenete trahydrofolate reductase and DNA methyltransferase 1 in human [32]. Anthocyanin and anthocyanidin extracts from blueberry can inhibit the proliferation and trigger the apoptosis of B16-F10 cells [33]. Blueberry consumption (400 mg daily) can reduce tumor size significantly in mice, which inhibited the proliferation of ovarian cancer cells by downregulating the levels of cyclooxygenase-1 and cyclooxygenase-2 [34].

### 3.2. Anti-Obesity and Functional Ingredients of Blueberry

Obesity is associated with the infiltration of monocytes into adipose tissue causing inflammation that is associated with metabolic abnormalities. Anthocyanins from blueberry dealcohol fermented beverages can inhibit insulin signaling in adipocytes, which induced insulin glucose uptake and reduced glycerol release in adipocytes [35] (see Table 1). Blueberry polyphenols can play an important role in inhibiting adipogenesis and cell proliferation [36] (see Table 1). Intake of blueberry leaf extract reduced body weight (20%) and improved insulin resistance as well as prevent obesity in mice with a high-fat diet [70], however blueberry extract possess a therapeutic tool against comorbidities related with obesity [71]. Expression of fatty acid synthase was significantly decreased in the adipose tissue of the liver and abdomen when wild blueberry consumption was increased [72]. Obesity and diabetes are becoming a global health problem; however, freeze-dried blueberry powder can treat and prevent obesity-related chronic diseases [73].

### 3.3. Prevent Degenerative Diseases and Functional Ingredients of Blueberry

The blueberry has excellent antioxidant activity and α-glucosidase inhibition [74]. The antiproliferative and antioxidants from blueberry juices are associated with anthocyanins; blueberry can prevent many degenerative diseases with daily consumption [37] (see Table 1). Anthocyanins can reduce the reactive oxygen species (ROS) and xanthine oxidase-1 (XO-1) capacities, but strengthen superoxide dismutase (SOD) and heme oxygenase-1 (HO-1), as well as malvidin capacities [38]. Four cultivars (Elliott, Rubel, Rancocas, and Friendship) with high anthocyanins and polyphenol content of 45 blueberries in Suwon have high antioxidant activity [39]. Chlorogenic acid concentration in lowbush blueberries is 0.44 mg/g, the phenolic acid mixture exerts it anti-inflammatory properties by controlling nuclear factor-κB activation and producing inflammatory cytokines at high dose [40]. Blueberry (fruit, pomaceas, and leaves) is one of the best sources of polyphenols with antioxidants, however its antioxidant activity is higher than that of rutin, especially, polyphenols in rabbiteye blueberry leaves has the highest antioxidant activity [41].

### 3.4. Anti-Inflammatory and Functional Ingredients of Blueberry

Dietary anthocyanins ameliorate inflammation and obesity as well as obesity-associated chronic diseases [75]. Phenolic acids (6.6%), flavonoids (12.9%), and procyanidins (2.7%) in blueberry extract have antibacterial and anti-inflammatory activity [42] (see Table 1). Blueberry with plentiful anthocyanins can increases anti-inflammatory cytokines and reduces oxidative stress in acute ingestion [43] (see Table 1). Blueberry is used as a functional food for preventive chronic inflammation; however, the anti-inflammatory effect of malvidin-3-glucoside is better than that of malvidin-3-galactoside [44]. Wild blueberry consumption has an overall anti-inflammatory effect in the metabolic syndrome [76]. The antibacterial and anti-inflammatory effects protect the oral keratinocyte barrier as well as neutralize leukotoxin properties of blueberry proanthocyanidins which could be promising candidates as novel therapeutic agents [45].

### 3.5. Protective Vision and Functional Ingredients of Blueberry

Total polyphenol and anthocyanin contents with improving mammal vision in wild Chinese blueberries were 0.60 and 0.18%, 13 anthocyanins were identified, especially malvidin (blue color) glycosylated with hexose (C_6_H_12_O_6_) or pentose that accounted for more than 46% [77]. The consumption of anthocyanin blueberry hastened the recovery of visual acuity after photobleaching [46] (see Table 1). Anthocyanins in berries contribute to eye health, especially cyanidin-3-glucoside in blueberry, is a preventive functional food for the prevention of retinal diseases [47]. Blueberry polyphenols have the retinal protective activity against light-induced retinal injury in eyes [48] (see Table 1).

### 3.6. Protective Liver and Functional Ingredients of Blueberry

Hepatitis C virus belongs to the genus *Hepacivirus*, which infects about 170 million people in the world and causes high rates of chronic hepatitis (>75%), however oligomeric proanthocyanidin in blueberry leaves can inhibit RNA expression of the hepatitis C virus [78]. Diets containing blueberries markedly reversed the acrylamide-induced alterations in liver exerting strong antioxidant activities, significantly alleviating the DNA damage in liver cells [79]. Pretreatment of blueberry anthocyanin can inhibit reactive oxygen species formation, prevent mitochondrial damage and even dysfunction in mice liver [28]. The anthocyanidins and anthocyanins in blueberry can inhibit the four enzymes catalytic activities of human hepatocytes and microsomes, but cyanidin-3-*O*-rhamnoside and two glycosides of delphinidin markedly inhibited CYP450 [49] (see Table 1). Redox response against a toxic selenite of rat brain and liver dose was associated with blueberry polyphenols, especially chlorogenic acid and flavonols that take on significant antioxidant protective effects [50] (see Table 1). Blueberry extract can be used as a therapeutic agent in combating Cd(II)-induced tissue injury of mouse liver, and its protective effect including antioxidative and anti-inflammatory properties with metal-chelating capacity [80]. The hepatoprotective effect of blueberry and chitosan significantly decreased liver arginase activity and ornithine levels, as well as increased nitric oxide and glutathione levels [81]. Regulating histone acetylation for anthocyanins from blueberries could improve liver function and liver fibrosis indexes in rats with hepatic fibrosis, which genes promote apoptosis and inhibit the effect of antihepatic fibrosis [51].

### 3.7. Heart Disease Prevention and Functional Ingredients of Blueberry

Blueberry anthocyanins eased cyclophosphamide-induced cardiac injury and improved oxidative stress and cardiomyocyte apoptosis [52] (see Table 1). Blueberry pomace with abundant procyanidins possesses protective effects against diabetes, obesity, and coronary heart disease; however, extrusion processing can increase monomer and dimers of procyanidin [53]. Blueberry polyphenols prevent adult cardiomyocyte hypertrophy and cell death associated with norepinephrine [54] (see Table 1).

### 3.8. Antidiabetic Properties and Functional Ingredients of Blueberry

Wild blueberry consumption is associated with glucose metabolism in metabolic syndrome obese rats [82]. The metabolism of blueberry anthocyanins lightens the endothelial dysfunction of lipid toxicity and lessens vascular complications associated with diabetes [55] (see Table 1). Blueberry can lighten vascular complications in diabetes as some of its metabolites can restore cell surface glycosaminoglycans and attenuate endothelial inflammation [83]. Oxidative stress plays a key role in diabetes and its complications; however, a blueberry dietary regime can treat type 1 diabetics [84]. Anthocyanin-rich wild Chinese blueberry can protect β-cells against glucolipotoxicity and prevent diabetes [56]. Blueberries prevent glucose intolerance and hepatic steatosis which are associated with gene expression of hepatic fatty acid oxidation [85]. Adipose inflammation promotes insulin resistance and other complications, however, dietary blueberry can combat obesity-associated pathology [86]. The anthocyanin-rich blueberry can temper the postprandial glucose response, and its implications for cognitive and type 2 diabetes research [57]. Blueberry supplementation improves component changes in the gut microbiota, related with improving general inflammation and insulin signaling in high-fat-diet rats [87].

### 3.9. Improving Brain Health and Functional Ingredients of Blueberry

Whole fresh blueberry reduced apoptosis and eased histopathological findings of d-galactose-treated rat brain [88]. The l-galactose pathway was major route of ascorbic acid biosynthesis and its higher expression levels are associated with five genes [guanosine diphosphate-mannose-3′,5′-epimerase (GME), guanosine diphosphate-l-galactose phosphorylase (GGP), l-galactono-1,4-lactone dehydrogenase (GLDH), monodehydroascorbate reductase (MDHAR), and dehydroascorbate reductase (DHAR)] in blueberry fruits [89]. Blueberry anthocyanin diets of animals can prevent irradiation by reducing oxidative stress and inflammation, improving neuronal signaling and protective neuronal functioning with exposure high energy particles [90]. Anthocyanins in blueberry improve cognitive function promoting brain perfusion and activation in healthy older adults [58,59] (see Table 1). The antidepressant-like effects of blueberry extract might be mediated by the controlling monoaminergic systems and glucocorticoids, which are due to neuroprotective effects and antagonism of the 5-HT receptor [91]. Cyaniding-3-*O*-galactoside in blueberry can protect the central nervous system and can increase cognitive and behavioral function in the ageing process through enhancing the antioxidation capacity and altering stress signaling [92]. Blueberries anthocyanins and ethylacetate fraction of blueberry leaf were related with increased neuronal signaling in the brain, mediating memory function and glucose disposal as well as delay neurodegeneration [60,93]. The dogs proportion of cognitive improvements was higher in dogs fed with polyphenol extract from blueberry [94]. Phenolics from blueberry can reduce gastrointestinal infection for patients of cerebral venous thrombosis by improving antidepressant activity via upregulation of miR-155-regulated brain-induced neurotrophic factor [61] (see Table 1). Elevated neural activation with blueberry shows neurocognitive benefit in this at-risk population [95]. The neurocognitive benefits of blueberries are mostly due to anthocyanin, eicosapentaenoic acid, and docosahexaenoic acid [62]. Six berry extracts especially blueberry anthocyanins have the scavenging free radical trapping reactive carbonyl, glycossylation resistance, anti-Aβ fibrillation, and microglial neuroprotective effects against Alzheimer’s disease [96].

### 3.10. Lung-Protective Properties and Functional Ingredients of Blueberry

The anthocyanidin (cyanidin, malvidin, peonidin, petunidin, and delphinidin) mixture of blueberry can inhibit the growth and invasive nature of lung cancer cells, which has very good therapeutic potential in non-small-cell lung cancer treatment, especially preventive future recurrence and metastasis [2] (see Table 1). Water soluble compounds of blueberry enzymatic hydrolysis possess strong antioxidant activity against H_2_O_2_-induced cell damage of lung fibroblast [97].

### 3.11. Strong Bones and Functional Ingredients of Blueberry

Bone mineral density in the distal epiphysis was markedly different between blueberry and blackberry, and their anthocyanin composition will affect bone turnover [63]. The consumption of blueberries diet can prevent ovariectomy-induced bone loss in rats, its molecular mechanisms are promoted by myosin production which stimulates osteoblast differentiation and reduces interstitial cell senescence [98] (see Table 1). Blueberry can prevent bone loss to increase bone mineral density during bone metabolism, which may be due to lowered femoral mRNA levels of alkaline phosphatase and collagen type I [99].

### 3.12. Enhance Immunological Effects and Functional Ingredients of Blueberry

Blueberries alleviate immunomodulation, lighten oxidative stress, and inflammation in adults of the metabolic syndrome [100]. Polysaccharides (100 mg/kg·d) from blueberry can inhibit the tumor growth rate approximately 73.4%, which could act as a good immunomodulator [64] (see Table 1). Blueberry can not only protect hepatocytes from oxidative stress and modulate the immune function of T-cells in mice, as well as induces the expression of three genes [101], but also enhances hepatic immunity in rats [102]. The immunoregulatory effect of flavonoids of blueberry leaves modulate the suppression of the TNF-α via and NF-κB signal pathways [65]. The obesity caused by a high-fat diet (HFD) can disrupt systemic immune function having deleterious impacts on T-cell hyperplasia and splenocyte immune abilities, however, dietary blueberry can improve T-cell and systemic immune function against HFD-obesity [103].

### 3.13. Prevent Cardiovascular Diseases and Functional Ingredients of Blueberry

Polyphenols in blueberry can regulate the vascular remodeling and improving the endothelial function both in smoker and in nonsmoker subjects [66]. Fermented blueberries with antihypertension properties can reduce the risk of cardiovascular diseases [104]. Blueberry-derived aglycone phenolic acids can lure Nrf2-controled antioxidant proteins in vascular endothelial cells [67]. Daily blueberry consumption can reduce blood pressure, aortic systolic pressure, arterial stiffness, diastolic pressure, and certain cancers in human due to increased nitric oxide production [105,106] (see Table 1). Dietary flavonoid-rich blueberries are very effective in regulating primary hypertension, due to suppression of soluble angiotensin-converting enzyme activity [68]. Wild blueberries possess protective properties against cardiovascular disease [107].

### 3.14. Blueberries can Improve Cognitive Decline, Functional Ingredients of Blueberry

Polyphenols have the beneficial effect of ameliorating cognitive decline in aging adults, especially, a polyphenol-rich extract from blueberry increases the memory ability of people with a large variety of cognitive impairments [69] (see Table 1). Three-month intervention with 100 mg blueberry extract can improve episodic memory performance and reduce cardiovascular risk factors over 6 months in an aged population [108]. The consumption of blueberry extracts regulates protein expression (e.g., dynamin 1) which is related to improved cognitive dysfunction in the hippocampus of APP/PS1 transgenic mice [109].

## 4. Major Mechanisms and Structural Activity of Blueberry Compounds for Preventive Chronic Disease

### 4.1. Anthocyanins Mechanism and its Structural Activity

**Figure 1:**
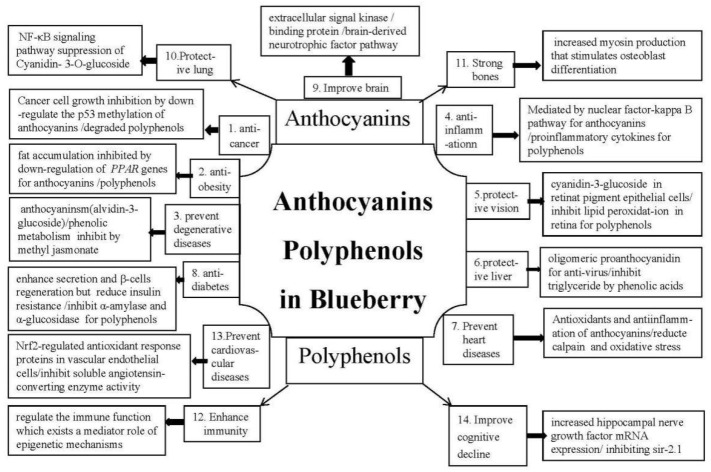
Possible mechanisms of functional ingredients in blueberry for preventive chronic disease.

Anthocyanins are a class of water-soluble flavonoids commonly consumed in the diet. Blueberry anthocyanins contribute to anticancer, anti-obesity, prevent degenerative diseases, anti-inflammation, protective properties for vision and liver, prevent heart diseases, antidiabetes, improve brain function, protective properties for the lung, and strong bones (see Table 1 and Figure 1). Blueberry is a super functional food with lots of health benefits due to its high levels of flavonoids. Flavanone 3-hydroxylase is a key regulatory enzyme of the flavonoid biosynthetic pathway for anthocyanin synthesis; anthocyanins are accumulated in fruits, whereas flavonols are found in leaves and stems [110]. Blueberry and malvidin for STAT-3 inhibitors in the oral cancer cell line SCC131, which regulates downstream targets that influence cell proliferation and apoptosis by abolishing the JAK/STAT-3 signaling pathway in a hamster model of oral oncogenesis [111]. Blueberry extracts exert anti-AML (acute myeloid leukemia) effects against myeloid leukemia cell lines, especially provoked by extracellular signal-regulated kinase (Erk) and protein kinase B (Akt) regulation within the leukemia stem cell subpopulation [112]. Blueberry anticancer effects associated with antioxidant and anti-inflammation, but controlling proliferation by regulating signal transduction. Functional ingredients of blueberry restrain the growth and metastasis of breast cancer cells by modulating the PI3K/AKT/NF-κB pathway [113]; the expression of caspase-9 and cytochrome c as well as the downregulation of the methylation of p53 of blueberry anthocyanins suppressed the proliferation and induced G2/M cell arrest and oral cancer cells apoptosis [114]. Blackberry anthocyanins and blueberry anthocyanins can ameliorate diet-induced obesity by alleviating oxidative stress and inflammation and quickening energy expenditure [115]. Blueberry peel extracts reduced weight and fat accumulation in obese rats by the downregulation of three genes and the reduction of phospho-Akt adipogenic factor in obese cells [116]. The antioxidant capacity of malvidin-3-glucoside in blueberry was stronger than that of malvidin-3-galactoside, glycosides improved the antioxidant capacity of malvidin to a great extent [38]. Eight-hundred-sixty-two transcription factors of 1236 transcripts in blueberry are involved in the antioxidant biosynthesis pathway of which about 92 expressed genes regulating anthocyanins in fruit during ripening [117]. The anti-inflammation mechanism of blueberry with malvidin-3-glucoside and malvidin-3-galactoside in endothelial cells was regulated by the nuclear factor-kappa B pathway [44]. Cyanidin-3-glucoside in blueberry has an ortho hydroxyl group for B ring with a multitude of eye-protective effects in human retinal pigment epithelial cells [47]. Blueberry anthocyanins have a protective effect on eye health which restrains the development of age-related macular degeneration; the anthocyanins reduced the growth factor levels of vascular endothelial cells and activated Akt-signal pathways [118]. Blueberry anthocyanins can prevent diabetic retinopathy and protect human retinal capillary endothelial cells via antioxidant and anti-inflammatory mechanisms [119]. Blueberry anthocyanins have protective effects on CCl4 induced hepatic fibrosis, which associated to decrease ROS producing sources and oxidative damage, as well as the influence of pro-inflammatory cytokines, inhibit the activity of hepatic stellate cells, downregulation TIMP1, PCNA, Col-III, α-SMA, and upregulation MMP-9 [120]. The action mechanism of oligomeric proanthocyanidin in blueberry leaves for resistant hepatitis C virus is an inhibitor of hnRNP A2/B1 [78]. The blueberry probiotics could antagonize the nonalcoholic fatty liver disease via the phosphor-Janus kinase-1/phosphor-signal transducer and activator of transcription 3 signaling pathway [121]. The reno-protective effects of blueberry inhibit TLR4; however, the TLR4-MAPK signaling pathway is very important to renal structural injury and dysfunction in MetS [122]. The mechanisms of blueberry anthocyanins for preventive heart diseases was due to its anti-inflammation and antioxidation [52]. The antidiabetic effects of blueberry are attributed to anthocyanins and polyphenols, however their mechanism is an increase in insulin secretion for pelargonidin, reduction of insulin resistance for cyanidin-3-glucoside, and enhancement of β-cells regeneration [123]. The neuroprotective mechanism of anthocyanins in blueberry involves the regulation of the signal transduction processes and gene expression in the brain [124]; anthocyanins and flavanols from blueberries enter the brain and can prevent changes in spatial working memory in aged animals, which are associated with flavonoids on the extracellular signal-related kinase and cAMP-response element-binding protein as well as brain-derived neurotrophic factor pathway [125]. Cyaniding-3-*O*-galactoside in blueberry can improve hippocampal neuron survival, increase hippocampal phosphorylated extracellular pegulated protein kinases expression, prevent pyramidal cell layer damage, increase superoxide dismutase activity, and reduce malondialdehyde content in the brain [92]. Cyanidin and cyanidin-containing products from blueberry can prevent oxidative stress in neuronal cells by strengthening mitochondrial activity and reducing intracellular ROS output as well as lipid peroxidation induced by H_2_O_2_, especially upregulated by CAT and SOD activities [126]. Cyanidin-3-*O*-glucoside, with lung-protective effects on rats, is the most active anthocyanin in the blueberry, which relates the suppression of the NF-κB signaling pathway [127]. Flavonoids, especially saponarin and lutonarin, have a preventive and therapeutic role for chronic diseases and have an antidiabetic effect; regulate blood pressure; protect liver; have antidepressant activity, anticancer and anti-inflammatory activity, antioxidant and hypolipidemic effects prevent cardiovascular diseases, and have antihypoxia and anti-fatigue effects [128].

### 4.2. Polyphenols Mechanism and its Structural Activity

Polyphenols (including quercetin) contribute to anticancer and anti-obesity activity, prevent degenerative diseases, have anti-inflammation activity, have protective effects on vision and liver, prevent heart disease, have antidiabetes properties, enhance immunity, prevent cardiovascular diseases, and improve cognitive decline (see Table 1 and Figure 1). The quercetin in onion has preventive properties for chronic disease that include anticancer, cardiovascular and heart diseases, anti-inflammation, anti-obesity, antidiabetes, antimicrobial activities, neuroprotective, and immunological effects [129]. Wild blueberry polyphenols were degraded to some phenolic compounds after colonic fermentation, which have lower antioxidant activities and cancer cell growth inhibition effects [18]. Blueberry leaf extract, especially the polyphenol component, showed inhibited adipocyte differentiation and decreased lipid accumulation by downregulating gene expression of peroxisome proliferator-activated receptor (*PPAR*) and acetyl CoA carboxylase (*ACCase*) and upregulating the mRNA expression of adiponectin compared with the high-fat diet group [70]. Blueberry with antioxidative properties and even health effects are primarily due to anthocyanins and methyl jasmonate, however methyl jasmonate can promote gene expression and phenolic metabolism in blueberry [130]. The anti-inflammatory mechanism of blueberry polyphenols was revealed by regulating the pro-inflammatory cytokines balance of interleukin-1β (-6 and -12), especially blueberry polyphenols (10–200 μg/mL) had the most significant suppression of these three genes [22]. Blueberry polyphenols, especially quercetin, inhibit lipid peroxidation of unsaturated fatty acids in the retina so that antioxidants can nourish the eyes [131]. Wild Chinese blueberries can prevent nonalcoholic fatty liver disease, majorly due to the action of phenolic acids inhibiting triglyceride accumulation in HepG2 cells [131]. Blueberry polyphenols prevent heart diseases, which are associated with the reduction of calpain activity and oxidative stress [54]. Blueberry extract has hypoglycemic activity exerted by promoting the expression of GLUT-2 and PPARγ and controlling the inflammatory pathway; based on interaction between caffeoylquinic acid derivatives and quercetin glycosides [132]. Blueberry polyphenols and its fermented beverages can inhibit α-amylase and α-glucosidase of starch-degrading for type 2 diabetes management [133], however polyphenols can regulate the immune function which exists as a mediator role of epigenetic mechanisms [134]. The polyphenols slow aging in organisms from worm and fly to rodent and human [135]. Blueberries can protect the kidney against AKI by inhibiting toll-like receptor 4 and its succedent effect on inflammation and oxidative stress [136]. Polyphenols from blueberry, such as flavan-3-ols, anthocyanins, and resveratrol can modulate brain synaptic plasticity and cognitive processes especially preventing older memory deficits, based on increased mRNA expression of hippocampal nerve growth factor [137]. Functional polyphenols, such as α-synuclein, have a therapeutic role for preventing protein aggregation related with neurodegeneration; however, Alaskan blueberries can alleviate pathologies of protein misfolding diseases by inhibiting sir-2.1 [138]. The memory protection of highbush blueberry vinegar can activate the brain-induced neurotrophic factor/cAMP response element restraining protein/serine-threonine kinase signaling [139].

## 5. Conclusions

Blueberry is one of the best functional foods, its total output was 629,720 tons in 2016 and its major distributors in the world are USA, Chile, Spain, and China. Blueberry is rich in flavonoids, polyphenols, phenolic acids, pyruvic acid, chlorogenic acid, and others. Blueberry is rich in anthocyanidins and polyphenols that are currently recommended among Chinese and international markets. Anthocyanidins in blueberry include cyanidin (red, violet, and blue change with pH), pelargonidin (orange), malvidin (blue), petunidin (dark-red, purple), and delphinidin (blue, blue-red); its polyphenols include 3-glucoside/arabinoside/galactoside-based polymers of delphinidins, petunidins, peonidins, malvidins, and cyanidins; its antioxidant properties are major total phenolic, quinic, chlorogenic acids, and rutin. The systematic review summarized describes the mechanisms of anthocyanidin and polyphenols from blueberry for preventive chronic diseases.

Blueberry anthocyanins have a lot of health benefits for human beings, which include anticancer, anti-obesity, prevent degenerative diseases, anti-inflammation, protective vision, protective liver, prevent heart diseases, antidiabetes, improve brain, and protective lung. Anthocyanins mechanism of blueberry for preventive chronic diseases are major as follows: The anticancer effect of anthocyanins is mediated by abolishing the JAK/STAT-3 signaling pathway and modulating the PI3K/AKT/Erk/Akt/P53/NF-κB pathway. The anti-obesity effect of anthocyanins is mediated by the downregulation of three genes and the reduction of the phospho-Akt adipogenic factor. Prevent degenerative diseases associated with stronger antioxidant capacity of malvidin-3-glucoside in blueberry; anti-inflammation of anthocyanins regulated by the nuclear factor-kappa B pathway; protective vision of cyanidin-3-glucoside in retinal pigment epithelial cells can reduce the growth factor of vascular endothelial cell and activated Akt-signaling, protective liver of anthocyanins is down-regulation TIMP1, PCNA, Col-III, α-SMA, and upregulation MMP-9 as well as an inhibitor of hnRNP A2/B1; prevent heart diseases of anthocyanins associated with anti-inflammation and antioxidation; the antidiabetic effect of anthocyanins increases the insulin secretion and reduces insulin resistance as well as enhances β-cell regeneration. The protective effect of anthocyanins on the brain is mediated by the extracellular signal-related kinase and cAMP-response element-binding protein, as well as brain-derived neurotrophic factor pathway and gene expression in brain. The protective effect of anthocyanins with relation to the lung is mediated by the suppression of the NF-κB signaling pathway.

Blueberry polyphenols have been shown to be beneficial to human health, and have beneficial properties which include anticancer, anti-obesity, prevention of degenerative diseases, anti-inflammation, protective effect on vision and liver, prevention of heart disease, antidiabetes, enhanced immune function, prevention of cardiovascular diseases, and improved cognitive decline. The mechanisms of polyphenols for the prevention of chronic diseases are as follows. Anticancer: polyphenols inhibit cancer cell growth by degraded polyphenols. Anti-obesity: polyphenols downregulate *PPAR* and *ACCase* gene expression and upregulate the mRNA expression. The prevention of degenerative diseases is associated with the gene expression and phenolic metabolism. The anti-inflammatory properties of polyphenols are mediated by the regulation of the pro-inflammatory cytokine balance and suppression of three genes. The protective vision property of polyphenols is mediated by the inhibition of lipid peroxidation in the retina. The protective liver property of polyphenols is mediated by the inhibition of triglyceride accumulation in HepG2 cells. The preventative effect on heart disease is associated with a reduction in calpain activity and oxidative stress. The antidiabetic effect of polyphenols is mediated by the inhibition of α-amylase and α-glucosidase, promoting the expression of *GLUT-2* and *PPARγ* that control the inflammatory pathway. The enhanced immunity of polyphenols is affected via a mediator role of epigenetic mechanisms. The prevention of cardiovascular diseases by polyphenols is mediated by the inhibition of soluble angiotensin-converting enzyme activity. The improvement in cognitive decline of polyphenols in caused by an increase in mRNA expression of the hippocampal nerve growth factor and restraining protein/serine-threonine kinase signaling.

Although functional ingredients in blueberry for preventive chronic diseases seem a complicated task and functional foods for therapeutic interventions may open new avenues, a further search of scientific evidence, from more carefully controlled longer-duration observational, animal, and human trials, especially anthocyanins and polyphenols in blueberry extracts, demonstrates the health effects for the treatment of chronic diseases. Dietary blueberry is one of the most exciting potential natural sources for developing functional foods and novel drugs with improved efficiency, efficacy, and safety. These data supported that anthocyanidins and polyphenols from blueberry are very important for the prevention of chronic diseases.

This review provides useful information for future research, especially anthocyanidins and polyphenols for the prevention or treatment of chronic diseases. In addition, it will be interesting to understand the interconnection between functional ingredients in blueberry and preventive chronic diseases in clinical trials. Further studies unravel the therapeutic role of major compounds of other berries for chronic disease. This review may be used as a starting point for novel nutraceuticals, functional foods, and drugs for blueberry to improve the prognosis of chronic diseases.

## Figures and Tables

**Table 1:** Functional ingredients for preventive chronic disease in blueberry.

Chronic Disease	Functional Components	References
Anticancer	Anthocyanins, phenolic acids, pyruvic acid, pterostilbene	[15,25,26,27,28,29,30,33,34]
Anti-obesity	Anthocyanins, polyphenols	[35,36]
Prevent degenerative diseases	Anthocyanins, chlorogenic acid, polyphenols	[37,38,39,40,41]
Anti-inflammation	Anthocyanins, phenolic acids, flavonoids	[42,43,44,45]
Protective vision	Anthocyanins, polyphenols	[46,47,48]
Protective liver	Anthocyanins, polyphenols	[28,49,50,51]
Prevent heart diseases	Anthocyanins, procyanidin, polyphenols	[52,53,54]
Antidiabetes	Anthocyanins, polyphenols	[35,55,56,57]
Improve brain	Anthocyanins, phenolics	[58,59,60,61,62]
Protective lung	Anthocyanins	[2]
Strong bones	Anthocyanins	[63]
Enhance immunity	Polysaccharide, polyphenols	[64,65]
Prevent cardiovascular diseases	Phenolic acids, flavonoid	[66,67,68]
Improve cognitive decline	Polyphenols	[69]
